# Supplementary material for: Embryonic and post-embryonic development in the parasitic copepod Ive ptychoderae (Copepoda: Iviidae): Insights into its phylogenetic position
Source: PLoS One. 2023 Mar 7;18(3):e0281013. doi: 10.1371/journal.pone.0281013 (PMC9990918; doi:10.1371/journal.pone.0281013)

PLOS ONE

Embryonic and post-embryonic development in the parasitic copepod Iveptychoderae (Copepoda: Iviidae): insights into its phylogenetic position

--Manuscript Draft--

**Manuscript Number:**	PONE-D-22-29055
**Article Type:**	Research Article
**Full Title:**	Embryonic and post-embryonic development in the parasitic copepod Iveptychoderae (Copepoda: Iviidae): insights into its phylogenetic position
**Short Title:**	Larval development of a parasitic copepod in acorn worms
**Corresponding Author:**	Jr-Kai Yu, Ph.D.<br>Academia Sinica<br>Taipei, TAIWAN
**Keywords:**	Parasitic copepod, embryonic development, larval stages, phylogeny
**Abstract:**	Parasitic copepods are frequently discovered in many marine animals, and they exhibit great species diversity with remarkable morphological adaptations to their parasitic lifestyle. Similar to their free-living relatives, parasitic copepods usually develop through complex life cycle, but they eventually transform into a modified adult form with much reduced appendages. Although the life cycle and distinct larval stages have been described in a few species of parasitic copepods, particularly those infecting commercially valuable animals, very little is known about the developmental process of the species that transformed into extremely simplified adult body plan with almost no trace of appendages. This paucity also causes some difficulties when investigating the taxanomy and phylogeny of this kind of parasitic copepods. Here we describe the embryonic development and a series of sequential larval stages of a parasitic copepod, Ive ptychoderae, which is a vermiform endoparasite living inside the hemichordate acorn worms. We devised laboratory regimes that enable us raising large quantity of embryos and free living larvae, and obtaining post-infested I. ptychoderae samples from the host tissues. Using defined morphological landmarks, the embryonic development of I. ptychoderae can be categorized into eight stages (1-, 2-, 4-, 8-, 16-cell stages, blastula, gastrula, and limb bud stages) and the post-embryonic development comprises six larval stages (2 naupliar and 4 copepodid stages). Based on the comparisons of morphological characters in the nauplius stage, our results provide evidence to support that the Ive-group is more closely related to the Cyclopoida, which represents one of the two major clades that contain many highly transformed parasitic copepods. Thus, our new results help to resolve the problematic phylogenetic position of the Ive-group in previous study based on analysis using 18S rDNA sequences. Combining with more molecular data, future comparative analyses on the morphological features of copepodid stages in a broad range of parasitic copepods will further refine our understanding of the phylogenetic relationships of these animals.
**Order of Authors:**	Yu-Rong Cheng
	Ching-Yi Lin
	Jr-Kai Yu, Ph.D.
**Additional Information:**	
**Question**	**Response**
**Financial Disclosure**<br><br>Enter a financial disclosure statement that describes the sources of funding for the work included in this submission. Review the submission guidelines for detailed requirements. View published research	JKY was supported by intramural funding from the Institute of Cellular and Organismic Biology (https://icob.sinica.edu.tw/Eng) and the Career Development Award from Academia Sinica, Taiwan (98-CDA-L06)(https://www.sinica.edu.tw/en), and the grant from the Ministry of Science and Technology, Taiwan (101-2923-B-001-004-MY2)(https://www.nstc.gov.tw/?l=en). The funders play no role in the study design, data collection and analysis, decision to publish, or preparation of the manuscript.

Powered by Editorial Manager® and ProduXion Manager® from Aries Systems Corporation

articles from *PLOS ONE* for specific examples.<br><br>This statement is required for submission and **will appear in the published article** if the submission is accepted. Please make sure it is accurate.<br><br>**Unfunded studies**<br>Enter: *The author(s) received no specific funding for this work.*<br><br>**Funded studies**<br>Enter a statement with the following details:<br>• Initials of the authors who received each award<br>• Grant numbers awarded to each author<br>• The full name of each funder<br>• URL of each funder website<br>• Did the sponsors or funders play any role in the study design, data collection and analysis, decision to publish, or preparation of the manuscript?<br>• **NO** - Include this sentence at the end of your statement: *The funders had no role in study design, data collection and analysis, decision to publish, or preparation of the manuscript.*<br>• **YES** - Specify the role(s) played.<br><br>* typeset	
**Competing Interests**<br><br>Use the instructions below to enter a competing interest statement for this submission. On behalf of all authors, disclose any competing interests that could be perceived to bias this work—acknowledging all financial support and any other relevant financial or non-financial competing interests.<br><br>This statement is required for submission and **will appear in the published article** if the submission is accepted. Please make sure it is accurate and that any funding sources listed in your Funding Information later in the submission form are also declared in your Financial Disclosure statement.	NO authors have competing interests

View published research articles from *PLOS ONE* for specific examples.<br><br>**NO authors have competing interests**<br><br>Enter: *The authors have declared that no competing interests exist.*<br><br>**Authors with competing interests**<br><br>Enter competing interest details beginning with this statement:<br><br>*I have read the journal's policy and the authors of this manuscript have the following competing interests: [insert competing interests here]*<br><br>* typeset	
**Ethics Statement**<br><br>Enter an ethics statement for this submission. This statement is required if the study involved:<br><br>• Human participants<br>• Human specimens or tissue<br>• Vertebrate animals or cephalopods<br>• Vertebrate embryos or tissues<br>• Field research<br><br>Write "N/A" if the submission does not require an ethics statement.<br><br>General guidance is provided below. Consult the submission guidelines for detailed instructions. **Make sure that all information entered here is included in the Methods section of the manuscript.**	The collection and use of invertebrate deuterostome animals for scientific research in JKY laboratory was reviewed by the Institutional Animal Care & Use Committee, Academia Sinica, and a waiver of ethic approval was granted (Case number: 16-04-956). The experimental procedures for handling the acorn worm P. flava were approved by the Institutional Biosafety Committee, Academia Sinica (permit number: BSF0418-00003976).

Powered by Editorial Manager® and ProduXion Manager® from Aries Systems Corporation

**Format for specific study types**<br><br>**Human Subject Research (involving human participants and/or tissue)**<br>• Give the name of the institutional review board or ethics committee that approved the study<br>• Include the approval number and/or a statement indicating approval of this research<br>• Indicate the form of consent obtained (written/oral) or the reason that consent was not obtained (e.g. the data were analyzed anonymously)<br><br>**Animal Research (involving vertebrate animals, embryos or tissues)**<br>• Provide the name of the Institutional Animal Care and Use Committee (IACUC) or other relevant ethics board that reviewed the study protocol, and indicate whether they approved this research or granted a formal waiver of ethical approval<br>• Include an approval number if one was obtained<br>• If the study involved *non-human primates*, add *additional details* about animal welfare and steps taken to ameliorate suffering<br>• If anesthesia, euthanasia, or any kind of animal sacrifice is part of the study, include briefly which substances and/or methods were applied<br><br>**Field Research**<br><br>Include the following details if this study involves the collection of plant, animal, or other materials from a natural setting:<br>• Field permit number<br>• Name of the institution or relevant body that granted permission	
**Data Availability**<br><br>Authors are required to make all data underlying the findings described fully available, without restriction, and from the time of publication. PLOS allows rare exceptions to address legal and ethical concerns. See the PLOS Data Policy and FAQ for detailed information.	Yes - all data are fully available without restriction

A Data Availability Statement describing where the data can be found is required at submission. Your answers to this question constitute the Data Availability Statement and **will be published in the article**, if accepted.<br><br>**Important:** Stating 'data available on request from the author' is not sufficient. If your data are only available upon request, select 'No' for the first question and explain your exceptional situation in the text box.<br><br>Do the authors confirm that all data underlying the findings described in their manuscript are fully available without restriction?	
**Describe where the data may be found in full sentences. If you are copying our sample text, replace any instances of XXX with the appropriate details.**<br><br>• If the data are **held or will be held in a public repository**, include URLs, accession numbers or DOIs. If this information will only be available after acceptance, indicate this by ticking the box below. For example: *All XXX files are available from the XXX database (accession number(s) XXX, XXX.*).<br>• If the data are all contained **within the manuscript and/or Supporting Information files**, enter the following: *All relevant data are within the manuscript and its Supporting Information files.*<br>• If neither of these applies but you are able to provide **details of access elsewhere**, with or without limitations, please do so. For example:<br><br>*Data cannot be shared publicly because of [XXX]. Data are available from the XXX Institutional Data Access / Ethics Committee (contact via XXX) for researchers who meet the criteria for access to confidential data.*<br><br>*The data underlying the results presented in the study are available from (include the name of the third party*	All relevant data are within the manuscript

and contact information or URL).<br>• This text is appropriate if the data are owned by a third party and authors do not have permission to share the data.<br><br>* typeset	
Additional data availability information:	

Powered by Editorial Manager® and ProduXion Manager® from Aries Systems Corporation

Manuscript
Click here to access/download;Manuscript;Cheng et al_2022-1024.pdf

Short title: Larval development of a parasitic copepod in acorn worms

Embryonic and post-embryonic development in the parasitic copepod *Ive ptychoderae* (Copepoda: Iviidae): insights into its phylogenetic position

Yu-Rong Cheng[1] *, Ching-Yi-Lin[2], Jr-Kai Yu[2, 3] *

[1] Department of Fisheries Production and Management, National Kaohsiung University of Science and Technology, Kaohsiung, Taiwan,
[2] Institute of Cellular and Organismic Biology, Academia Sinica, Taipei, Taiwan
[3] Marine Research Station, Institute of Cellular and Organismic Biology, Academia Sinica, Yilan, Taiwan

* Corresponding authors
E-mail: yrcheng@nkust.edu.tw (YRC) and jkyu@gate.sinica.edu.tw (JKY)

Abstract

Parasitic copepods are frequently discovered in many marine animals, and they exhibit great species diversity with remarkable morphological adaptations to their parasitic lifestyle. Similar to their free-living relatives, parasitic copepods usually develop through complex life cycle, but they eventually transform into a modified adult form with much reduced appendages. Although the life cycle and distinct larval stages have been described in a few species of parasitic copepods, particularly those infecting commercially valuable animals, very little is known about the developmental process of the species that transformed into extremely simplified adult body plan with almost no trace of appendages. This paucity also causes some difficulties when investigating the taxonomy and phylogeny of this kind of parasitic copepods. Here we describe the embryonic development and a series of sequential larval stages of a parasitic copepod, *Ive ptychoderae*, which is a vermiform endoparasite living inside the hemichordate acorn worms. We devised laboratory regimes that enable us raising large quantity of embryos and free living larvae, and obtaining post-infested *I. ptychoderae* samples from the host tissues. Using defined morphological landmarks, the embryonic development of *I. ptychoderae* can be categorized into eight stages (1-, 2-, 4-, 8-, 16- cell stages, blastula, gastrula, and limb bud stages) and the post-embryonic development comprises six larval stages (2 naupliar and 4 copepodid stages). Based on the comparisons of morphological characters in the nauplius stage, our results provide evidence to support that the *Ive*-group is more closely related to the Cyclopoida, which represents one of the two major clades that contain many highly transformed parasitic copepods. Thus, our new results help to resolve the problematic phylogenetic position of the *Ive*-group in previous study based on analysis using 18S rDNA sequences. Combining with more molecular data, future comparative analyses on the morphological features of copepodid stages in a broad range of parasitic copepods will further refine our understanding of the phylogenetic relationships of these animals.

Introduction

Parasitic copepods exhibit various reductive tendencies, such as simplification and reduction of adult appendages [1-3]. This often poses a major obstacle to research on taxonomy and systematics of these animals, since classification of copepods is mainly based on the differences in morphology of adult females. It has been particularly challenging when dealing with the taxonomy and phylogeny of the two copepod orders, Poecilostomatoida and Cyclopoida, which include many species with highly transformed adults [4]. Traditionally, a cladistics phylogeny of ten copepod orders (Calanoida, Cyclopoida, Gelyelloida, Harpacticoida, Misophrioida, Monstrilloida, Mormonilloida, Platycopioida, Poecilostomatoida, and Siphonostomatoida) were proposed based on the morphological homologies in the body plan, segmentation and setation of appendages [3]. However, recent molecular analyses have shown some conflicting results with this phylogenetic scheme and questioned the validity of Poecilostomatoida [5, 6]. Nowadays, the order Cyclopoida is generally considered encompassing Poecilostomatoid families, as suggested by several phylogenetic studies [7-10].

In those parasitic copepods with transformed body plan or with extremely reduced appendages, their larvae may provide rich information of additional morphological, behavioral, and ecological characters. The embryogenesis and post-embryonic stages (including nauplius and copepodid stages) of copepods have been investigated by many authors [4, 11-20]. The morphological features of larval stages have been considered as useful characters for understanding the taxonomy and phylogeny [4, 18, 21-23]. Naupliar characters could represent plesiomorphic states and nauplii of closely related taxa are more likely to conserve synapomorphies than their adults [23]. To date, however, there are only a few parasitic copepods whose life cycle and larval stages have been thoroughly described (e.g. [13, 24-28]). This shortage is mainly due to the difficulty of obtaining live ovigerous females and their eggs for life cycle research. Therefore, for those problematic parasitic copepods, studies on their life cycle and larval morphology would not only advance our knowledge of their life histories, but also may provide key information for resolving their phylogeny.

Previously ~~our research group~~ identified a new species of parasitic copepod, *Ive ptychoderae*, living inside the acorn worm *Ptychodera flava* (Hemichordata,

Enteropneusta) collected from Penghu Islands in Taiwan [29]. The female of *I. ptychoderae* exhibits a highly transformed body plan (vermiform) which lacks apparent body segmentation and possesses ~~greatly~~ reduced appendages. Characterized by having a reduced maxilliped and five pairs of annular swellings along the body, *I. ptychoderae* can be distinguished from the two previously reported parasitic copepods in acorn worms [29].Those two endo-parasites had been described more than one hundred years ago: *Ive balanoglossi* was collected from *Glossobalanus minutus* Kowalevsky, 1866 in the Mediterranean Sea [30]; *Ubius hilli* was described [illegible] parasite of *Balanoglossus australiensis* Hill, 1894 in Australian waters [31]. These two monotypic genera ~~also~~ possess highly transformed body plan and reduced appendages, and their phylogenetic affiliations remain unsettled. Traditionally, the Pennellidae and Chondracathidae, had been used as convenient repositories for problematic parasitic copepods [32]. Due to the absence of clear external segmentation, *Ive* was originally grouped with Pennellidae by Mayer [30], but subsequently Kesteven [31] preferred to classify *Ive* and *Ubius* as members of the Chondracanthidae. However, due to the lack of definitive diagnostic features, eventually these two genera were not allocated to any family in the Copepoda and temporarily grouped together into the *Ive*-group by later researchers [32]. In our recent study, we attempted to investigate the phylogenetic position of the newly discovered *Ive ptychoderae* by combining the results from morphological features of adults and molecular data [29]. Our molecular phylogenetic analysis showed that *I. ptychoderae* is likely a member of the Poecilostomatoida, but its 18S rDNA sequence is so distinct that it cannot be associated with any copepod family with known 18S rDNA sequence. Therefore, a new family, Iviidae, was proposed then to accommodate both *Ive* and *Ubius*. Nevertheless, until now the *Ive*-group is still treated as problematic taxa and its phylogenetic position remains ambiguous.

In this study, we seek to examine the life cycle of *I. ptychoderae* and utilize the morphological characters of its larvae to improve our understanding of the phylogenetic affiliation of this parasitic copepod. The first naupliar stage of *I. balanoglossi* and *U. hilli* were described previously by Mayer [30] and Kesteven [31], respectively. Other than that, nothing is known about the embryonic and larval development in the *Ive*-group. Here, we describe the life cycle of *I. ptychoderae* from embryonic stages, nauplius I to copepodid IV larval stages, as well as the morphological features of these developmental stages. In comparison with other

copepods, we also discuss the phylogenetic implications of our results.

Materials and methods

Sample collection

Acorn worms *P. flava* were collected from the sandy beach at Chito, Penghu Islands, Taiwan (23°38'54.17"N; 119°36'14.40"E). To isolate the parasitic copepods, acorn worms were anesthetized with 0.2 M magnesium chloride in seawater for 15 minutes and the parasitic copepods were obtained from dissected cysts of hosts. The collection and use of invertebrate deuterostome animals for scientific research in JKY laboratory was reviewed by the Institutional Animal Care & Use Committee, Academia Sinica, and a waiver of ethic approval was granted (Case number: 16-04-956). The experimental procedures for handling the acorn worm *P. flava* were approved by the Institutional Biosafety Committee, Academia Sinica (Permit number: BSF0418-00003976).

Incubation experiments

Twenty ovigerous females were obtained and cleaned off the host mucus in 3.5 cm Petri dishes filled with filtered/sterilized sea water. The egg s[illegible] removed from the females were placed in the dishes and kept in water of 25±1 ppt salinity in the room temperature (about 25℃). The water in the dishes was changed and unhatched eggs and exuviae were removed with a pipet every 12 hrs.

Duration of embryonic and naupliar stages

For determining the duration of embryonic stages (1-, 2-, 4-, 8-cell, and so on), DAPI (4′, 6-diamidino-2-phenylindole) visualization of nuclei in fixed embryos were used to create reference stages of embryonic development. The embryonic development was monitored under ~~dissecting microscopy~~ at 0.5–1 h intervals and the duration time of embryonic development was documented. When the eggs developed into next step, they were fixed in 4 % Paraformaldehyde (PFA) for 12 h and then transferred into 96-well plates containing 100 μl of DAPI staining solution (5 μg/ml) at 4℃ in the dark overnight. The de-ionized (DI) water was used as a staining vehicle (creating an osmotic imbalance) to stress the egg chorion and to allow fluorescent

stain molecules into the eggs and nuclei. The stained eggs were washed by 1% phosphate-buffered saline with Tween® detergent (PBST) and examined for stages under epi-fluorescence microscopy. The higher resolution images were made under confocal microscopy with UV excitation at 405 [illegible]

In the case of mature eggs, approximately 3 d, over thousands nauplii (first naupliar stage) were obtained. The nauplii were maintained in seawater at same conditions a[illegible]eatments. The naupliar development was monitored under dissecting microscopy at 8 h intervals and the duration time was documented. When nauplii molted into infective stage (copepodid larvae), we reintroduced them to *P. flava* (re-infection study).

Re-infection study

In the re-infection study, ten individuals of *P. flava* were cut into 5 fragments (each about 1 cm) as surrogate hosts and infected by 10 copepodids, respectively. After infection, one to three infected acorn worms were sacrificed and examined e[illegible]y day. The acorn worms were placed in 3–5% ethanol solution for at least 4 h and washed by shaking. Then, the wash water was poured through a fine net (mesh size = 100 µm) and examined under a dissection microscope. The copepods were picked up by forceps and preserved in 70% ethanol.

Morphological studies

All pr[illegible]ved specimens (nauplii and copepodids) were in 85% lactic acid for at least 1 h, then were cleared before taking measurements and dissecting. The removed body parts and appendages were examined under a compound microscope [33]. All drawings were made with the aid of a drawing tube.

In this paper, the abbreviations NI and NII are used instead of nauplius I and II stages; the abbreviations CI to CIV are used instead of copepodid I to IV stages.

Results

Eggs and the duration of embryonic developments

The eggs of *I. ptychoderae* are usually laid in a pair of egg sacs carried by the female in the host. However, in some cases, the eggs are also shed free around the

female body. Each egg sac contains approximately 600 eggs (see [29]) and the eggs are generally 128 (120–142) µm in diameter (*n*=30, Fig 1).

The life history of *I. ptychoderae* was commenced from the time when the eggs were laid into the egg sacs. Based on our observation, the eggs in the same sac were non-synchronous in development. However, due to the nuclei and cleavages of eggs at all development stages were clearly visible, the embryonic development was easily separated into 8 stage categories: 1-, 2-, 4-, 8-, 16- cell stage, blastula, gastrula, and limb bud stages (Fig 1). The duration of embryogenesis totally spent about 70 h from 1 cell stage to limb bud stage, and limb bud stages accounted for more than half of the development time (37 h) (Fig 2).

Fig 1. The embryonic stages of *Ive ptychoderae*. (A) One-cell stage; (B) 2-cell stage; (C) 4-cell stage; (D) 8-cell stage; (E) 16-cell stage; (F) Blastula stage; (G) Gastrula stage; (H) Early limb bud stage; (I) Late limb bud stage. Scale bars: A = 0.5 mm; B–I = 0.05 mm.

Fig 2. The duration of embryonic stages (1-, 2-, 4-, 8-, 16- cell, blastula, gastrula, and limb bud stages) of *Ive ptychoderae*. The assigned time of each stage is indicated in parentheses.

Morphology of nauplius stages

The naupliar larvae hatched out from the mature eggs at the end of late limb bud stage. We made continuous observations of naupliar molting and identified two free-swimming naupliar stages and four copepodid stages (Figs 3–10). They are described separately as follows:

Fig 3. Images of Nauplius I stage larva of *Ive ptychoderae*; (A) Bright-field image, and (B) fluorescent image by confocal microscopy with nucleus staining (red). (a1) Antennule; (a2) antenna; (m) mandible; (*) yolky granules. Scale bar = 50 µm.

Nauplius I

Fig 4.

Body (Fig 4A) oval or pear-shaped, full of yolky granules (see Fig 3B), longer than wide, Average length 163 (161–166) μm and greatest width 105 (103–107) μm based on 10 specimens. Three pairs of swimming appendages: antennule, antenna, and mandible. Labrum (Fig 4A) curved prominence without ornamentation. Caudal armature (Fig 4A) represented by 1 pair of fine setae. Antennule (Figs 4A and 4B) uniramous, 1-segmented, armed with 1 proximoventral, 1 midventral and 2 unequal terminal setae. Antenna (Figs 4A and 4C) biramous, with exodopod slightly longer than endopod; coxa without masticatory process, separate from short basis; exopod 4-segmented, first segment elongated and fused with basis, each segment armed with 1 seta; endopod 2-segmented, first segment unarmed, indistinctly separate from basis, second segment armed with 2 plumose setae. Mandible (Figs 4A and 4D) biramous, coxa separate from basis, both coxa and basis unarmed. Exopod 5-segmented, each armed with 1 plumose seta except for first segment; first segment elongated, fused with basis. Endopod 1-segmented, armed with 1 proximoventral, 1 midventral, and 2 equal terminal setae.

Fig 4. Nauplius stage I of *Ive ptychoderae*. (A) Habitus, ventral; (B) Antennule; (C) Antenna; (D) Mandible. Scale bars: A = 0.05 mm; B–D = 0.025 mm.

Nauplius II

Fig 5.

Body (Fig 5A) as in NI but slightly slender, average length 160 (158–163) μm and greatest width 94 (92–97) μm based on 10 specimens. Labrum and caudal armature (Fig 5A) as in NI. Antennule (Figs 5A and 5B) 2-segmented, first segment with 2 rows of spinules and 3 equal setae; second segment with 3 rows of spinules, one sub-distal seta (largest), and 2 unequal terminal setae. Antenna (Figs 5A and 5C) as in NI, without ornamentation on coxa and basis; exopod distinctly 5-segmented, with 1 plumose seta at inner tip of segments 1-4 and 2 unequal smooth seta on fifth segment; endopod 2-segmented, first segment unarmed, second segment armed with 1 middle smooth seta and 2 terminal plumose setae. Mandible (Figs 5A and 5D) as in NI.

Fig 5. Nauplius stage II of *Ive ptychoderae*. (A) Habitus, ventral; (B) Antennule; (C) Antenna; (D) Mandible. Scale bars: A = 0.05 mm; B–D = 0.025 mm.

Morphology of copepodid stages

Definition of the copepodid stages was based on the examination of morphological characters of antennule, antenna, legs 1 and 3 under the light microscopy. From our own observations, we determined that the copepodid stages consist of 4 stages. They are described separately as follows:

Copepodid I

Fig 6.

Body (Fig 6A) with broad flattened prosome. Average length of 10 specimens measured 260 (248–268) μm (excluding seta) and greatest width 93 (91–95) μm. Prosome 2-segmented. Cephalothorax distinctly longer than wide, 130×93 μm; second prosomal somite wider than long, 68×33 μm. Urosome (Figs 6A and 6B) 3-segmented, from anterior to posterior 85 μm. Third segment longest, about 38 μm in length and 24 μm in width. Caudal ramus (Figs 6A–6C) small, about 15 × 11 μm, bearing with 6 setae, innermost terminal seta longest, consisting of thicker proximal part and slender distal flagellum. Surface of body unornamented (Fig 6A).

Antennule (Fig 6D) 4-segmented, with setal formula: 6, 4, 2+1 aesthetasc, and 6+1 aesthetasc, all setae naked. Antenna (Fig 6E) 4-segmented, consisting of coxobasis and 3 endopodal segments, each endopodal segments with 1 claw and 1 seta; exopod vestigial and unarmed.

Labrum (Fig 6F) with concave posterior margin and well developed rounded lateral lobes. Mandible, maxillule, and maxilliped absent.

Legs 1 and 2 (Figs 6G–6I) biramous, each ramus with 1-segmented exopods and endopods. Formula of spines (in Roman numerals) and setae (in Arabic numerals) as follows:

	Coxa	Basis	Exopod	Endopod
Leg 1	0-0	1-0	IV-3	III-4
Leg 2	0-0	1-0	IV-3	III-3

Legs 3 and 4 absent. Leg 5 (Fig 6A) reduced to minute process bearing a terminal seta and an adjacent dorsal seta.

Fig 6. Copepodid stage I of *Ive ptychoderae*. (A) Habitus, dorsal; (B) Urosome, dorsal; (C) Caudal ramus; (D) Antennule; (E) Antenna; (F) Labrum; (G) Leg 1; (H) Leg 2; (I) Endopod of leg 2. Scale bars: A = 0.05 mm; B–I = 0.025 mm.

Copepodid II

Fig 7.

Body plan (Figs 7A and 7B) as in CI, average length 244 (228–260) μm and greatest width 91 (89–93) μm based on 10 specimens. Caudal ramus (Fig 7B) as in CI.

Antennule (Fig 7C) armature and segmentation as in the previous stage, except for an aesthetasc instead of seta on second segment. Antenna (Fig 7D) 3-segmented, consisting of coxobasis and 2 endopodal segments; endopod first segment unarmed, second segment divisible into 3 parts: proximal part carrying 1 small claw; middle part armed with 1 seta and 1 claw; and distal part with 1 slender claw and 1 longest seta. Labrum as in CI. Mandible, maxillule, and maxilliped not present. Legs 1–5 as in the previous stage.

Fig 7. Copepodid stage II of *Ive ptychoderae*. (A) Habitus, dorsal; (B) Urosome, dorsal; (C) Antennule; (D) Antenna; Scale bars: A = 0.05 mm; B–D = 0.025 mm.

Copepodid III

Fig 8.

Body (Fig 8A) moderately slender. Average length 218 (206–228) μm and greatest width 73 (70–75) μm based on 9 specimens. Segmentation of body indistinct. Caudal ramus (Figs 8A and 8B) elongated, bearing 1 terminal longest seta (= innermost terminal seta in the previous stages), and 2 inner, marginal setae in addition to 2 setae on outer-lateral margin.

Antennule (Figs 8C and 8D) 4-segmented, setal formula: 6, 4, 3, and 3+1 aesthetasc. Antenna (Figs 8C and 8E) 2-segmented, basal segment largest, bearing patch of spinules on middle margin, second segment with 1 long seta with lamella, 2 small spiniform setae, and 2 claws.

Labrum (Fig 8C) projected posteriorly at both free corners. Mandible, maxillule, and maxilliped not present.

Legs 1 and 2 (Figs 8F and 8G) biramous, with 1-segmented exopod and endopod. Formula of spines (in Roman numerals) and setae (in Arabic numerals) as follows:

	Coxa	Basis	Exopod	Endopod
Leg 1	0-0	0-0	II-3	2
Leg 2	0-0	0-0	II-3	3

Legs 3 and 4 absent. Leg 5 (Fig 8A) reduced to minute process bearing a terminal seta and an adjacent small seta.

Fig 8. Copepodid stage III of *Ive ptychoderae*. (A) Habitus, dorsal; (B) Caudal ramus; (C) Antennal area and oral region, ventral; (D) Antennule; (E) Antenna; (F) Leg 1; (G) Leg 2. Scale bars: A = 0.05 mm; B–G = 0.025 mm.

Copepodid IV

Fig 9.

Body (Fig 9A) slender than CIII. Average length 856 (465–1175) μm and greatest width 164 (107–190) μm based on 10 specimens. Segmentation of body indistinct. Caudal ramus (Fig 9B) carrying 1 inner, 2 lateral, and 2 terminal tubercles.

Antennule (Fig 9C) short, unsegmented, divisible into 3 parts: proximal part carrying 2 unequal setae; middle part armed with 1 large and 2 small setae; distal region with 3 small tubercles. Antenna (Fig 9D) 3-segmented, first and second segments unarmed; third segment with 1 large claw and 1 pointed process.

Labrum (Fig 9E) projected posteriorly at both free corners. Mandible, maxillule, and maxilliped not present. Legs 1 and 2 as in adult (Figs 9F and 9G; see [29]). Legs 3–5 absent.

Fig 9. Copepodid stage IV of *Ive ptychoderae*. (A) Habitus, dorsal; (B) Caudal ramus; (C) Antennule; (D) Antenna; (E) Antennal area and oral region, ventral; (F) Leg 1; (G) Leg 2. Scale bars: A = 0.1 mm; B–G = 0.025 mm.

Adult

Since the morphology of adult stage had been reported in detail by Tung et al. [29], they are not necessary to be re-described again in the paper.

The duration of post-embryonic developments

In total, the duration of post-embryonic development lasted 38 d and included 6 stages, namely, NI and NII (free-swimming stage) and CI through CIV (infective stage) (Fig 10). The nauplii passed through 2 stages to become copepodid and underwent a single molt at every stage. The larvae of NI swam actively and were positively phototactic. However, the swimming activity decreased as the nauplii started to molt. Approximately 2 d, the NI molted into NII. The larvae of NII took an average 7 d for growing into the first infective stage (CI). The newly molted CI larvae were rather restless, wandering over the Petri dishes to search for a suitable microhabitat of host fragment by synchronously moving two pairs of legs. The duration of this stage to the next stage (CII) was relatively short (approximately 2 d) due to a few morphological changes. The differences between CI and CII were only occurred in the length/width ratio of prosome and armature of antennule and antenna. However, there were dramatic differences in morphology and behavior since CII stage. The duration from CII to CIII was comparatively long as well as the duration from CIII to CIV. Both phase lasted 7-9 d; consequently, it took about 2 weeks in total from CII to CIV. It should be mentioned that the duration of CIV lasted at least for 11 d until the end of our experiment. The structures of appendages are highly similar to adult (see [29]), but the body size is much small, ranging from 0.47 to 1.76 mm.

Fig 10. Duration of post embryonic stages of *Ive ptychoderae*. (1-2) naupliar stages; (I-IV) copepodid stages; (V) adult stage. The duration time (days) of each stage is provided and the number of copepod individuals at each developmental stage is indicated in parentheses. Dotted line indicates the end of incubator experiment.

Discussion

Copepods represent a diverse animal group with great morphological variations and versatile life cycle strategies [4, 34]. In this study, we described the life cycle of a

parasitic copepod *I. ptychoderae* and the morphological features characterizing each developmental stage in this species. These new results provide important information to suggest the phylogenetic affiliation of this parasitic copepod.

Ive ptychoderae exhibits a typical process of embryogenesis

In general, copepods usually require about 24–72 h from eggs to hatching [35-37]. Buttino et al. [38] developed a new staining protocol for the visualization of copepod embryos with the confocal laser scanning microscope. Since then, fluorescence probes have been used on the studies of copepod embryonic development [38, 39]. However, this approach has rarely been used in the parasitic copepods. Here, we show that the embryos of parasitic copepod can also be rapidly assessed using nucleus staining under fluorescent microscope (Fig 3). We estimated that the pre-hatching embryonic development of *I. ptychoderae* is approximately 70 h, which is in line with previous observations in copepod species. And we observed that the limb bud stages accounted for approximately half of the embryogenesis period (about 37 h). This is conceivable because organogenesis occurs in limb bud stages and takes more time to develop complex structures of the larva, so that the newly hatched nauplius can be equipped with necessary organ systems for the subsequent planktonic phase of its life cycle [40].

Ive ptychoderae reduces its naupliar and copepodid stages

The basic life cycle of symbiotic copepods encompasses two planktonic phases, the naupliar and copepodid stages, during which the animals may perform diverse behaviors including dispersal, infection, host switching, mating, and presumably, predator-avoidance [41-43]. In general, it has been known that symbiotic copepods typically have 12 post-embryonic stages including 6 nauplius, 5 copepodid stages, and then molt into adult stage [13-15, 17-19, 23, 34]. However, abbreviated life cycle is characteristic for some parasitic copepods, thus they are highly efficient in locating and infecting new hosts without needing long-distance larval dispersal [4, 14, 18, 19, 25, 26, 44]. We also observed the reduction of naupliar and copepodid stages in *I. ptychoderae*. Based on the morphological characters described in this study, the post-embryonic development of *I. ptychoderae* can be categorized into 6 (2 nauplius and 4 copepodid) stages from NI to CIV stage. Becasue adult *I. ptychoderae* is an endo-parasite with highly transformed body plan and reduced appendages, the exhibition of

a reductive life cycle is not unexpected. It may reflect its adaptation to the parasitism.

The morphological characters of *I. ptychoderae* nauplius larvae and their implications to the phylogenetic affiliation of this highly transformed parasitic copepod

Izawa [4] suggested that two types of naupliar forms were defined based on the mode of nutritional supply or egg size (the amount of yolk granules). The copepods eggs less than 120 μm may yield feeding nauplii (planktotrophic form) which usually have the basic six naupliar stages. On the other hand, the copepod eggs with large size may yield lecithotrophic nauplii (non-feeding form) and reduce in the number of naupliar stages. Our results showed that the eggs of *I. ptychoderae* were larger than 120 μm, and that ample yolk granules were observed inside the nauplius stage larvae (Fig. 3). This suggests that (1) the nauplii of *I. ptychoderae* are lecithotrophic form and do not feed during the nauplius stages, and (2) its naupliar stage may less than six stages. Lecithotrophic nauplii are common to most parasitic copepods belonging to the orders Cyclopoida and Siphonostomatoida [4, 16, 26, 32, 45]. The body shape of nauplii of *I. ptychoderae* are fundamentally oval or pear-shaped, which is also considered as a typical morphology in the siphonostomatoids and cyclopoids [4, 15, 25]. However, the fish-parasitizing siphonostomatoids have evolved a remarkable trait by converting the copepodid stages into a series of more effectively attached chalimus stages in their life cycles. Without recognizable chalimus stage, *I. ptychoderae* is not likely belonging to the order Siphonostomatoida and thus may be more closely related to the Cyclopoida.

Mayer [30] and Kesteven [31] described the first nauplius stage of *I. balanoglossi* and *U. hilli*, respectively. They provided a figure to show a whole animal in ventral view and the morphological information of appendages. Combining with the results from previous studies and our observations, the nauplii of *Ive*-group possess some common morphological features. For example, their bodies were full of yolky granules, lacked a masticatory process on the antennal coxa, and the labrums were reduced in size. However, the antenna of *Ive*-group with 2-segmented endopod in the nauplius stage (as in the Facetotecta and Cephalocarida) is a primitive character for distinguishing them from the existing orders of Copepoda. In addition, the exopod of antenna of *I. ptychoderae* is 4 segments in the first nauplius stage, but the generalized exopod is 5–6 segments in most copepod such as Siphonostomatoida,

Cyclopoida, Calanoida, and Harpacticoida. Nevertheless, it should be noted that the exopod of antenna may decrease in the typical number of segments in the nauplii of some parasitic copepods [25, 27, 46]. The other characters in the naupliar stage of *Ive*-group also provided some signs to distinguish them from other copepod orders. According to Izawa [4], two type of apical setation of the mandibular exopod (with 1 or 2 setae) are recognized in the nauplii of copepods. By using this feature, systematic positions of some problematic copepods are resolved. For example, the Antheacheridae, Mesoglicolidae, and Herpyllobiidae have been correctly accommodated in the Cyclopoida (= Poecilostomatoida) based on their nauplii with one seta type; the Sponginticolidae (treated as order uncertain) has been removed to Siphonostomatoida since it exhibited the two setae type in the nauplius stage. Thus, the presence of one apical seta on the mandibular exopod in the nauplius stage of *Ive*-group is a main character for distinguishing them from the species belonging to the Calanoida, Harpacticoida, and Siphonostomatoida (with 2 setae). The number of apical elements of the antennule in the first nauplius stage of *Ive*-group with only 2 setae (instead of 3 or 4 setae) is similar to that of ergasilids in the Cyclopoida. Indeed, *Ive*-group might be more closely related to *Antelichomolgus*-group (including Clausidiidae, Oncaeidae, Taeniacanthidae, Tegobomolochidae, and Bomolochidae) belonging to the Cyclopoida based on four combinations of naupliar characters [4]. However, species in the *Antelichomolgus*-group have 6 naupliar stages, while there are only 2 naupliar stages in *I. ptychoderae*. Moreover, the nauplii of *I. ptychoderae* with 1-segmented mandibular endopod and 5-segmented mandibular exopod are significantly different from the general form in the cyclopoids (with 2-segmented mandibular endopod and 4-segmented mandibular exopod). All in all, based on the morphological characters of naupliar stages, *I. ptychoderae* exhibited many unique characters.

Dahms [23] pointed out several reasons to explain why naupliar characters have thus far been widely neglected in the systematic and phylogenetic studies: (1) It is difficult to obtain the detailed information on naupliar characters, (2) the nauplii may provide fewer characters than later ontogenetic stages, (3) It may lack appropriate comparative data, (4) non-feeding naupli[illegible]ay lack phylogenetically valuable structures, and (5) conflicting evidence would be occurred in comparing the character states between adult and nauplius stages. In the case of *I. ptychoderae*, the reason 3 is the most critical factor to limit the resolution of our results. The life cycles of

crustacean parasite are largely unknown (1% of species) [19]. Taking sea lice for example, there are more than 450 caligid species but the complete life cycles are now known only for 17 species (3.8%) representing just three genera, *Caligus* Müller 1785 (12 species), *Lepeophtheirus* von Nordmann 1832 (four species) and *Pseudocaligus* Scott 1901 (= *Caligus*; one species) [14, 47-49]. Although only some comparative data, the nauplii of *I. ptychoderae* indeed exhibit many distinguishable characters from the major existing orders of Copepoda. For a definitive view of the phylogenetic relationships of *Ive*-group, further phylogenetic discussion cannot be made on the basis of the known nauplii only. More information on the nauplii of other related copepods is indispensable for addressing this issue, and the features on the copepodid stages must also be taken into consideration. In addition, future molecular phylogenetic analysis including related species, such as *I. balanoglossi* and *U. hilli*, would be highly welcomed.

Funding

This work was supported by intramural funding from the Institute of Cellular and Organismic Biology and the Career Development Award from Academia Sinica, Taiwan (98-CDA-L06), and the grant from the Ministry of Science and Technology, Taiwan (101-2923-B-001-004-MY2).

Competing interests

The authors have declared that no competing interests exist.

Acknowledgements

We would like to thank Che-Huang Tung and Sharon Horng for their assistance in the field.

Author Contributions

Conceived and designed the experiments: YRC and JKY. Performed the experiments: YRC and CYL. Analyzed the data: YRC. Wrote the manuscript: YRC and JKY. All authors have read and approved the final manuscript.

References

1. Ho JS. Why do symbiotic copepods matter? Hydrobiologia. 2001;453(1-3):1-7. doi: 10.1023/A:1013139212227. PubMed PMID: WOS:000172600200001.
2. Humes AG. How many copepods. Hydrobiologia. 1994;293:1-7. doi: 10.1007/Bf00229916. PubMed PMID: WOS:A1994QG90200002.
3. Huys R, Boxshall GA. Copepod evolution. London: Ray Society; 1991.
4. Izawa K. Studies on the phylogenetic implications of ontogenetic features in the poecilostome nauplii (Copepoda: Cyclopoida). Publ Seto Mar Biol Lab. 1987;32:151-217.
5. Kim J, Kim W. Molecular phylogeny of poecilostome copepods based on the 18S rDNA sequences. Kor J Biol Sci. 2000;4(3):257-61.
6. Song Y, Wang GT, Yao WJ, Gao Q, Nie P. Phylogeny of freshwater parasitic copepods in the Ergasilidae (Copepoda: Poecilostomatoida) based on 18S and 28S rDNA sequences. Parasitol Res. 2008;102(2):299-306. Epub 2007/10/18. doi: 10.1007/s00436-007-0764-8. PubMed PMID: 17940799.
7. Huys R, Fatih F, Ohtsuka S, Llewellyn-Hughes J. Evolution of the bomolochiform superfamily complex (Copepoda: Cyclopoida): New insights from ssrDNA and morphology, and origin of umazuracolids from polychaete-infesting ancestors rejected. Int J Parasitol. 2012;42(1):71-92. doi: 10.1016/j.ijpara.2011.10.009. PubMed PMID: WOS:000300468900007.
8. Huys R, Llewellyn-Hughes J, Conroy-Dalton S, Olson PD, Spinks JN, Johnston DA. Extraordinary host switching in siphonostomatoid copepods and the demise of the Monstrilloida: Integrating molecular data, ontogeny and antennulary morphology. Mol Phylogenet Evol. 2007;43(2):368-78. doi: 10.1016/j.ympev.2007.02.004. PubMed PMID: WOS:000246918800002.
9. Huys R, Llewellyn-Hughes J, Olson PD, Nagasawa K. Small subunit rDNA and Bayesian inference reveal Pectenophilus ornatus (Copepoda incertae sedis) as highly transformed Mytilicolidae, and support assignment of Chondracanthidae and Xarifiidae to Lichomolgoidea (Cyclopoida). Biol J Linn Soc. 2006;87(3):403-25. doi: 10.1111/j.1095-8312.2005.00579.x. PubMed PMID: WOS:000235587900005.
10. Khodami S, Mercado-Salas NF, Tang D, Arbizu PM. Molecular evidence for the retention of the Thaumatopsyllidae in the order Cyclopoida (Copepoda) and establishment of four suborders and two families within the Cyclopoida. Mol Phylogenet Evol. 2019;138:43-52. doi: 10.1016/j.ympev.2019.05.019. PubMed PMID: WOS:000475550000004.
11. Anandan P, Krishnamurthy R, Altaff K. Studies on different stages of post embryonic development of cyclopoid copepod Apocyclops dengizicus. Int J Curr Microbiol App Sci. 2013;2(2):20-7.
12. Chullasorn S, Dahms HU, Iwasaki N, Kangtia P, Ferrari FD, Jeon HJ, et al. Naupliar Development of an Ancorabolid, Paralaophontodes sp (Copepoda: Harpacticoida) Sheds Light on Harpacticoid Evolution. Zool Stud. 2012;51(3):372-82. PubMed PMID: WOS:000304891000009.
13. Do TT, Kajihara T, Ho JS. The life history of *Pseudomyicola spinosus* (Raffaele and Monticelli, 1885) from the blue mussel, *Mytilus edulis galloprovincialis* in Tokyo Bay, Japan, with notes on the production of atypical male. Bull Ocean Res Inst Univ Tokyo. 1984;17:1-65.
14. Hamre LA, Eichner C, Caipang CMA, Dalvin ST, Bron JE, Nilsen F, et al. The Salmon Louse *Lepeophtheirus salmonis* (Copepoda: Caligidae) life cycle has

only two Chalimus Stages. Plos One. 2013;8(9). doi: ARTN e73539 10.1371/journal.pone.0073539. PubMed PMID: WOS:000326240100049.

15. Itoh H, Nishida S. Naupliar stages of *Hemicyclops japonicus* (Copepoda: Poecilostomatoida) reared in the laboratory. J Crustacean Biol. 1997;17(1):162-73. doi: 10.2307/1549470. PubMed PMID: WOS:A1997WH61800017.
16. Ivanenko VN, Arbizu PM, Stecher J. Lecithotrophic nauplius of the family Dirivultidae (Copepoda; Siphonostomatoida) hatched on board over the Mid-Atlantic Ridge (5 degrees S). Mar Ecol-Evol Persp. 2007;28(1):49-53. doi: 10.1111/j.1439-0485.2006.00142.x. PubMed PMID: WOS:000245546200006.
17. Kim IH. Copepodid stages of *Critomolgus anthopleurus* (Copepoda, Poecilostomatoida, Rhynchomolgidae). J Crustacean Biol. 2003;23(3):558-67. doi: 10.1651/C-2375. PubMed PMID: WOS:000184295900005.
18. Ohtsuka S, Madinabeitia I, Yamashita H, Venmathi Maran BA, Suárez-Morales E, Ho JS. Planktonic phases in symbiotic copepods: a review. Bull Southern California Acad Sci. 2018;117(2):104-19.
19. Williams EHJ, Bunkley-Williams L. Life cycle and life history strategies of parasitic crustacea. In: Smit NJ, Bruce NL, Hadfield KA, editors. Parasitic crustacea: state of knowledge and future trends. 3. New York: Springer, Cham; 2019. p. 179-266.
20. FD F, HU D. Post-embryonic development of the Copepoda. Crustac Monogr. 2007;8:1-232. doi: 10.1163/ej.9789004157132.i-230.
21. Dahms HU. Naupliar development of *Paraleptastacus brevicaudatus* Wilson, 1932 (Copepoda, Harpacticoida, Cylindropsyllidae). J Crustacean Biol. 1990;10(2):330-9. doi: 10.2307/1548491. PubMed PMID: WOS:A1990DC25200013.
22. Dahms HU. Metamorphosis between naupliar and copepodid Phases in the Harpacticoida. Philos T Roy Soc B. 1992;335(1274):221-36. doi: 10.1098/rstb.1992.0020. PubMed PMID: WOS:A1992HH24900005.
23. Dahms HU. Postembryonic apomorphies proving the monophyletic status of the Copepoda. Zool Stud. 2004;43(2):446-53. PubMed PMID: WOS:000222853500031.
24. Gonzalez L, Carvajal J. Life cycle of *Caligus rogercresseyi*, (Copepoda : Caligidae) parasite of Chilean reared salmonids. Aquaculture. 2003;220(1-4):101-17. doi: 10.1016/S0044-8486(02)00512-4. PubMed PMID: WOS:000182099100009.
25. Ho JS, Cheng YR, Lin CL. The development of *Parabrachiella lata* (Song & Chen, 1976), a lernaeopodid copepod parasitic on the black porgy, *Acanthopagrus schlegelii* (Bleeker, 1854), of Taiwan. Crustaceana. 2007;80(1):47-66. doi: 10.1163/156854007779696505. PubMed PMID: WOS:000244788400005.
26. Izawa K. On the development of parasitic Copepoda IV. Ten species of poecilostome cyclopoids, belonging to Taeniacanthidae, Tegobomolochidae, Lichomolgidae, Philoblennidae, Myicolidae, and Chondracanthidae. Publ Seto Mar Biol Lab. 1986;31:81-162.
27. Piasecki W. Life cycle of *Tracheliastes maculatus* Kollar, 1835 (Copepoda, Siphonostomatoida, Lernaeopodidae). Wiad Parazytol. 1989;35(3):187-245. Epub 1989/01/01. PubMed PMID: 2728456.
28. Piasecki W, Mackinnon BM. Life cycle of a sea louse, *Caligus elongatus* von Nordmann, 1832 (Copepoda, Siphonostomatoida, Caligidae). Can J Zool. 1995;73:74-82. doi: 10.1139/z95-009.

29. Tung CH, Cheng YR, Lin CY, Ho JS, Kuo CH, Yu JK, et al. A new copepod with transformed body plan and unique phylogenetic position parasitic in the acorn worm *Ptychodera flava*. Biol Bull. 2014;226(1):69-80. doi: 10.1086/BBLv226n1p69. PubMed PMID: WOS:000333256700007.
30. Mayer P. Ein neuer parasitischer copepode. Mitt Zool Stn Neapel. 1879;1:515-21.
31. Kesteven HL. A new endoparasitic copepod: morphology and development. Proc Linn Soc N S W. 1913;37:673-88.
32. Boxshall GA, Halsey SH. An introduction to copepod diversity. London: Ray Society; 2004.
33. Humes AG, Gooding RU. A method for studying the external anatomy of copepods. Crustaceana. 1964;43(2):238-40. doi: 10.1163/156854064X00650.
34. Ferrari FD, Dahms HU. Post-embryonic development of the Copepoda. Crustac Monogr. 2007;8:1-232. doi: 10.1163/ej.9789004157132.i-230.
35. Ianora A. Copepod life history traits in subtemperate regions. J Marine Syst. 1998;15(1-4):337-49. doi: 10.1016/S0924-7963(97)00085-7. PubMed PMID: WOS:000074876100038.
36. Miralto A, Guglielmo L, Zagami G, Buttino I, Granata A, Ianora A. Inhibition of population growth in the copepods *Acartia clausi* and *Calanus helgolandicus* during diatom blooms. Mar Ecol Prog Ser. 2003;254:253-68. doi: 10.3354/meps254253. PubMed PMID: WOS:000183887600022.
37. Poulet SA, Ianora A, Miralto A, Meijer L. Do diatoms arrest embryonic development in copepods. Mar Ecol Prog Ser. 1994;111(1-2):79-86. doi: 10.3354/meps111079. PubMed PMID: WOS:A1994PB96700009.
38. Buttino I, Santo MD, Ianora A, Miralto A. Rapid assessment of copepod (*Calanus helgolandicus*) embryo viability using fluorescent probes. Mar Biol. 2004;145(2):393-9. doi: 10.1007/s00227-004-1317-7. PubMed PMID: WOS:000223098200019.
39. Zirbel MJ, Miller CB, Batchelder HP. Staging egg development of marine copepods with DAPI and PicoGreen (R). Limnol Oceanogr-Meth. 2007;5:106-10. doi: 10.4319/lom.2007.5.106. PubMed PMID: WOS:000247293300001.
40. Strathmann RR, Staver JM, Hoffman JR. Risk and the evolution of cell-cycle durations of embryos. Evolution. 2002;56(4):708-20. PubMed PMID: WOS:000175614400006.
41. Huys R. Copepoda. In: Martin JW, Olesen J, Hoeg JT, editors. Atlas of Crustacean Larvae. Maryland: John Hopkins University Press; 2014. p. 144-63.
42. Kearn GC. Leeches, lice and lampreys: a natural history of skin and gill parasites of fishes. Dordrecht: Springer; 2004.
43. Maran BAV, Suarez-Morales E, Ohtsuka S, Soh HY, Hwang UW. On the occurrence of caligids (Copepoda: Siphonostomatoida) in the marine plankton: a review and checklist. Zootaxa. 2016;4174(1):437-47. doi: 10.11646/zootaxa.4174.1.27. PubMed PMID: WOS:000385249700025.
44. Ivanenko VN, Ferrari FD, Smurov AV. Nauplii and copepodids of *Scottomyzon gibberum* (Copepoda : Siphonostomatoida : Scottomyzotidae, a new family), a symbiont of *Asterias rubens* (Asteroidea). P Biol Soc Wash. 2001;114(1):237-61. PubMed PMID: WOS:000168465000021.
45. Paterson NF. External features and life cycle of *Cucumaricola Notabilis* nov. gen. et. sp., a copepod parasite of the Holothurian, Cucumaria. Parasitology. 1958;48(3-4):269-90. doi: 10.1017/S0031182000021247. PubMed PMID: WOS:A1958WX55600005.
46. Piasecki W, Kuźmińska E. Developmental stages of *Achtheres percarum*

(Crustacea: Copepoda), parasitic on European perch, *Perca fluviatilis* (Actinopterygii: Perciformes). Acta Ichthyol Piscat. 2007;(37):187-245.

47. Madinabeitia I, Nagasawa K. Chalimus stages of *Caligus latigenitalis* (Copepoda: Caligidae) parasitic on blackhead seabream from Japanese Waters, with discussion of terminology used for developmental stages of caligids. J Parasitol. 2011;97(2):221-36. doi: 10.1645/Ge-2615.1. PubMed PMID: WOS:000290950500008.
48. Maran BAV, Moon SY, Ohtsuka S, Oh SY, Soh HY, Myoung JG, et al. The caligid life cycle: new evidence from *Lepeophtheirus elegans* reconciles the cycles of *Caligus* and *Lepeophtheirus* (Copepoda: Caligidae). Parasite. 2013;20. doi: ARTN 1510.1051/parasite/2013015. PubMed PMID: WOS:000319676700001.
49. Ohtsuka S, Takami I, Maran BAV, Ogawa K, Shimono T, Fujita Y, et al. Developmental stages and growth of *Pseudocaligus fugu* Yamaguti, 1936 (Copepoda: Siphonostomatoida: Caligidae) host-specific to Puffer. J Nat Hist. 2009;43(29-30):1779-804. doi: 10.1080/00222930902993757. PubMed PMID: WOS:000269193500003.

Figure 1

Click here to access/download;Figure;Figure1.tif

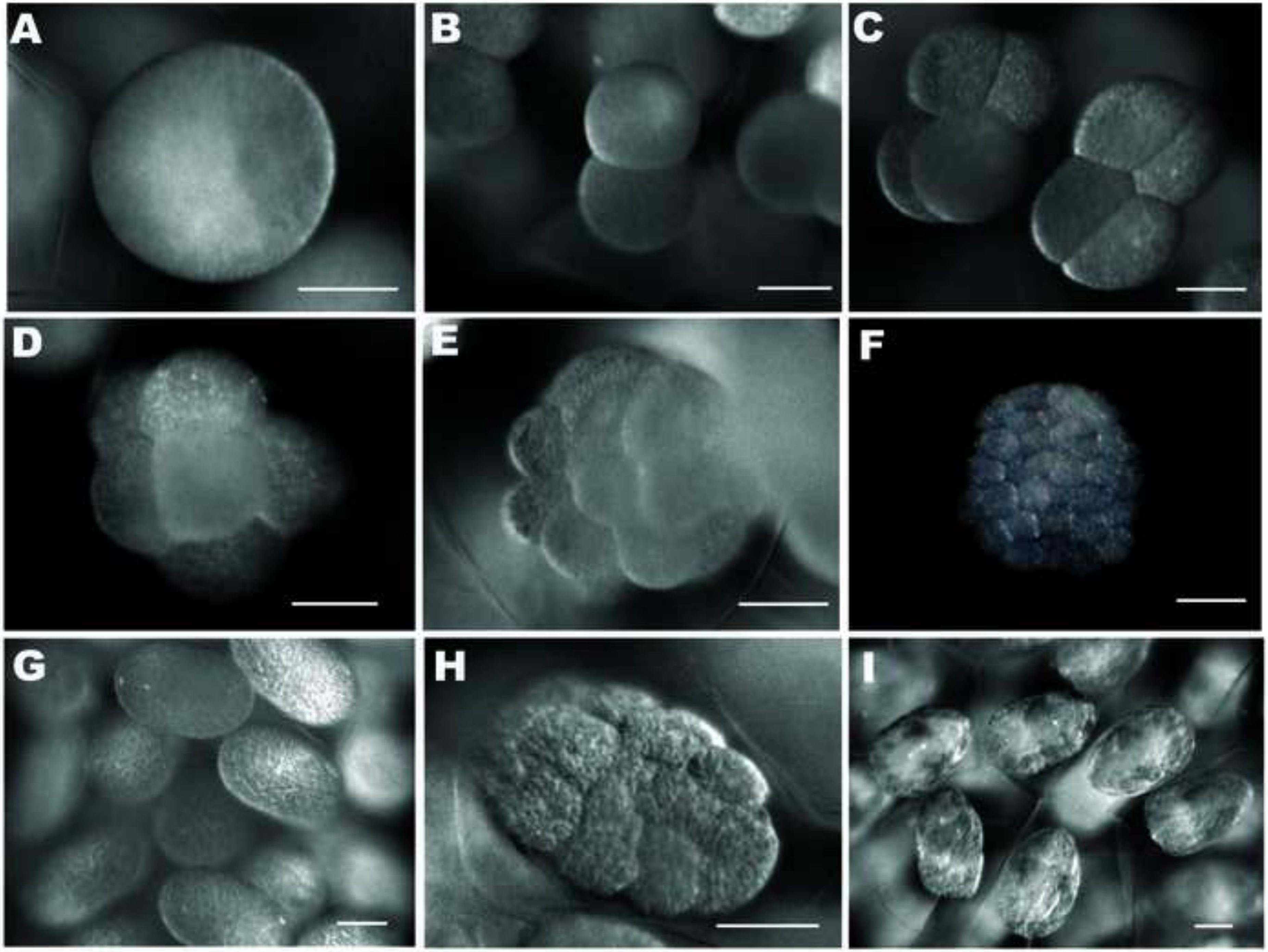

Click here to access/download;Figure;Figure2.pdf

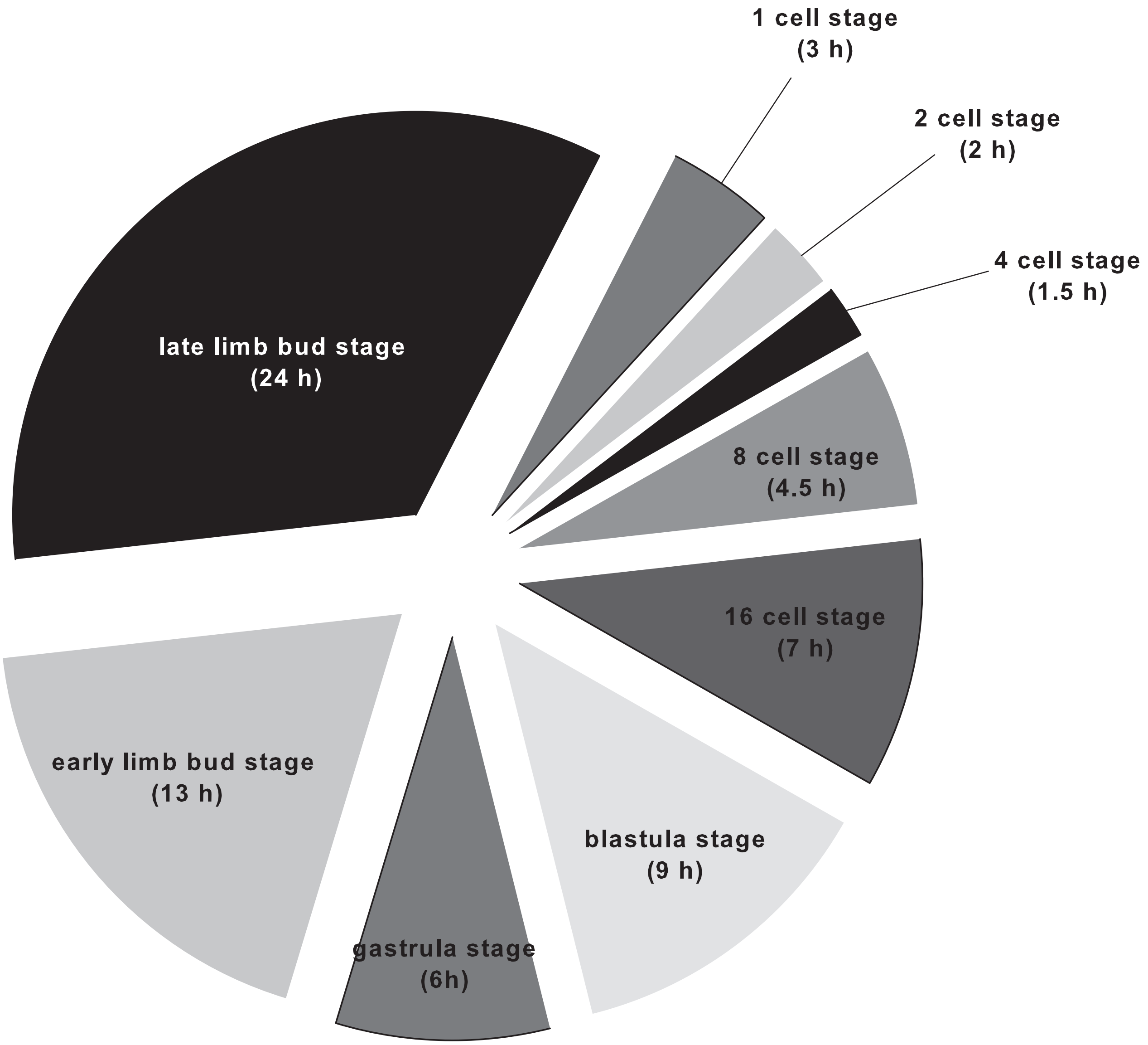

Figure 3

Click here to access/download;Figure;Figure3.tiff

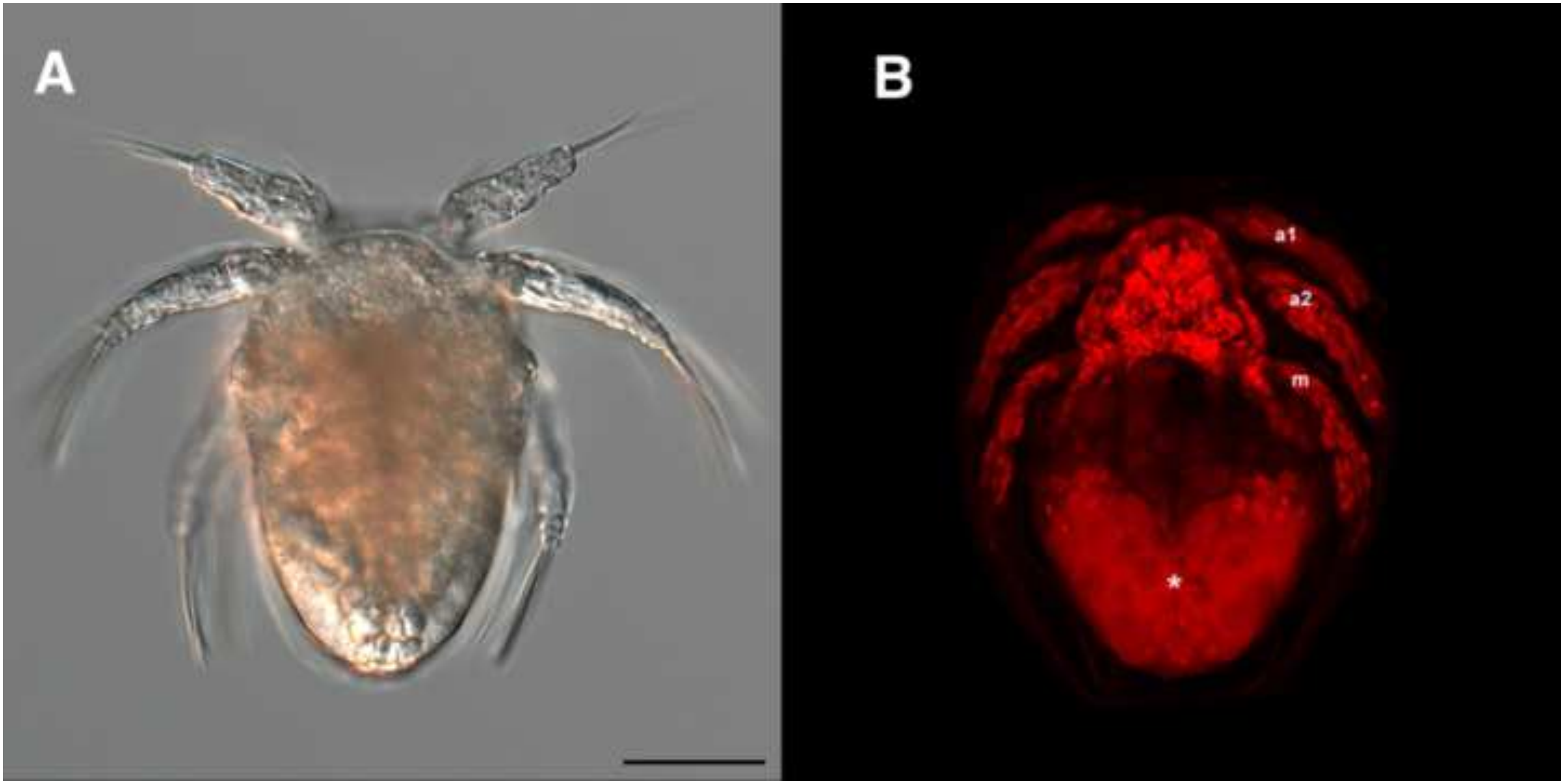

 Click here to access/download;Figure;Figure4.tif

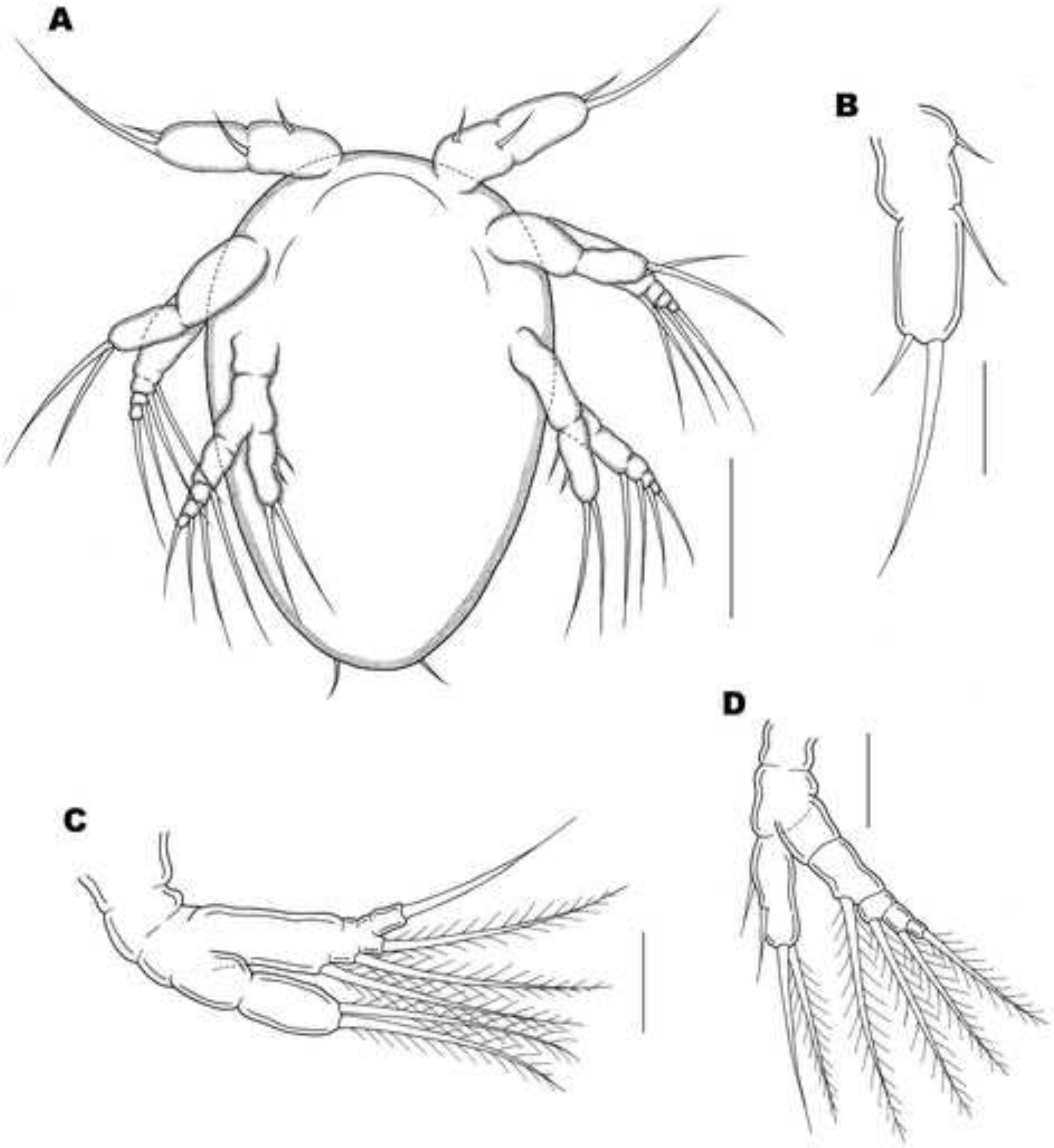

Figure 5

Click here to access/download;Figure;Figure5.tif

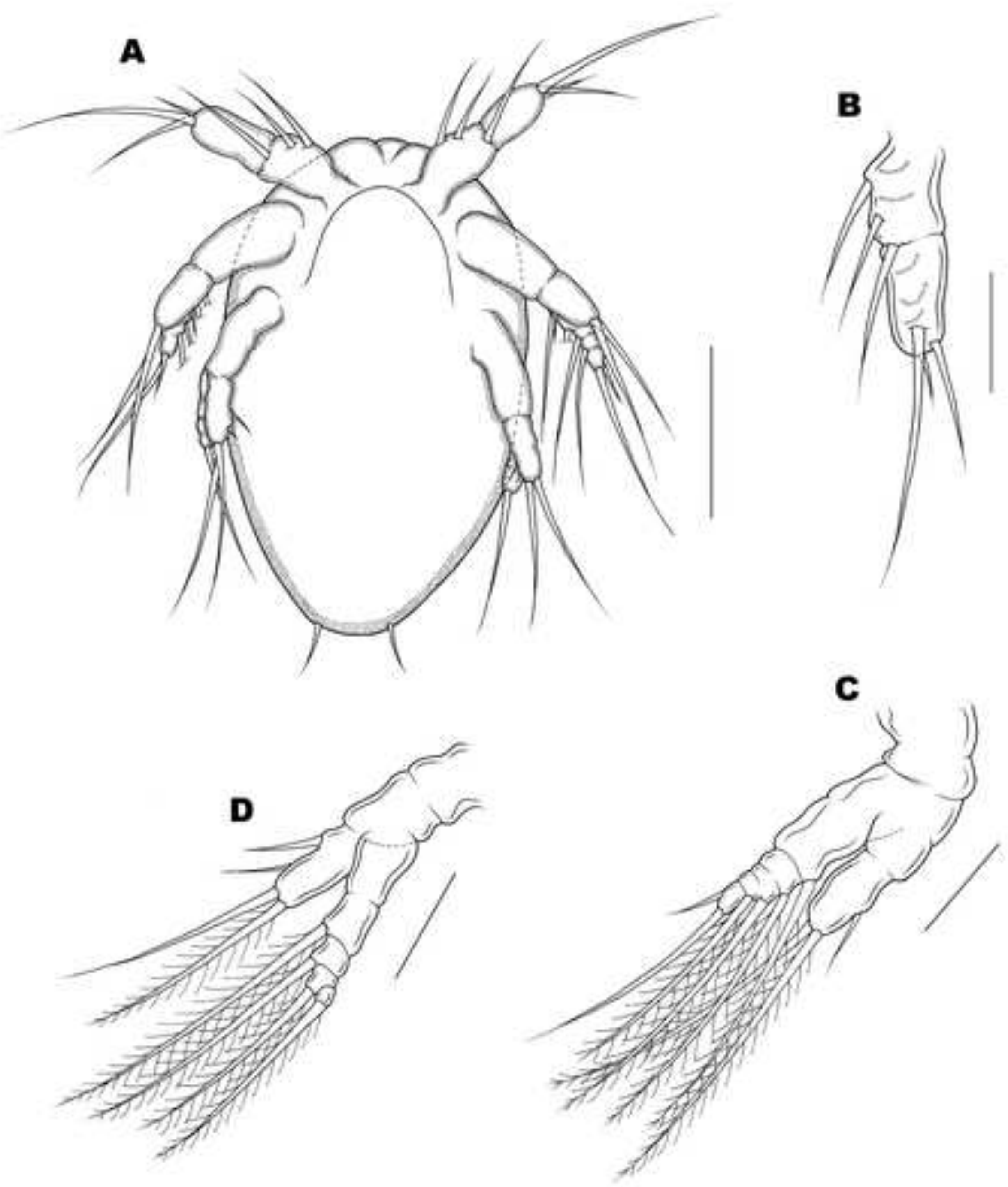

Figure 6

Click here to access/download;Figure;Figure6.tif

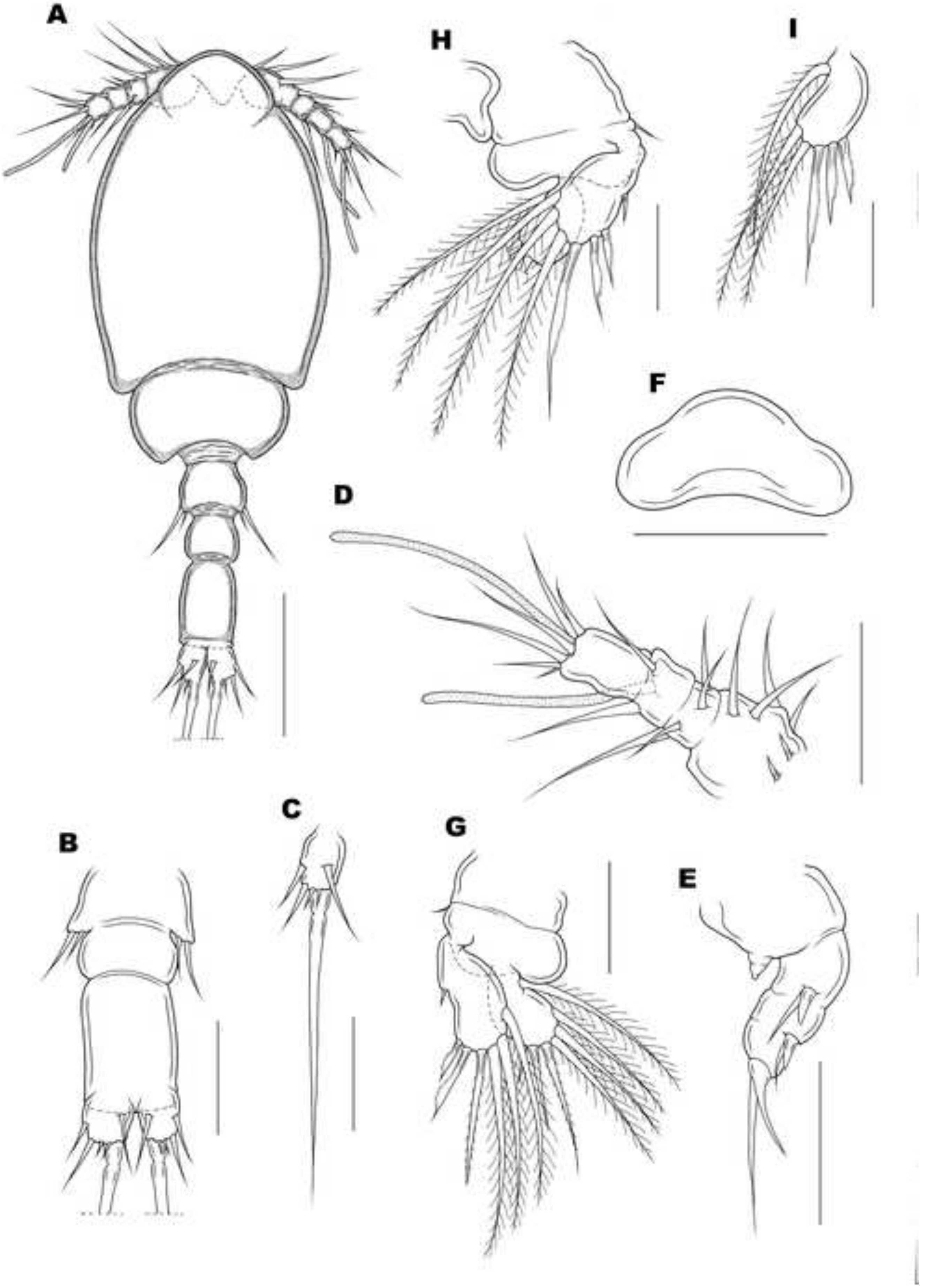

Figure 7

Click here to access/download;Figure;Figure7.tif

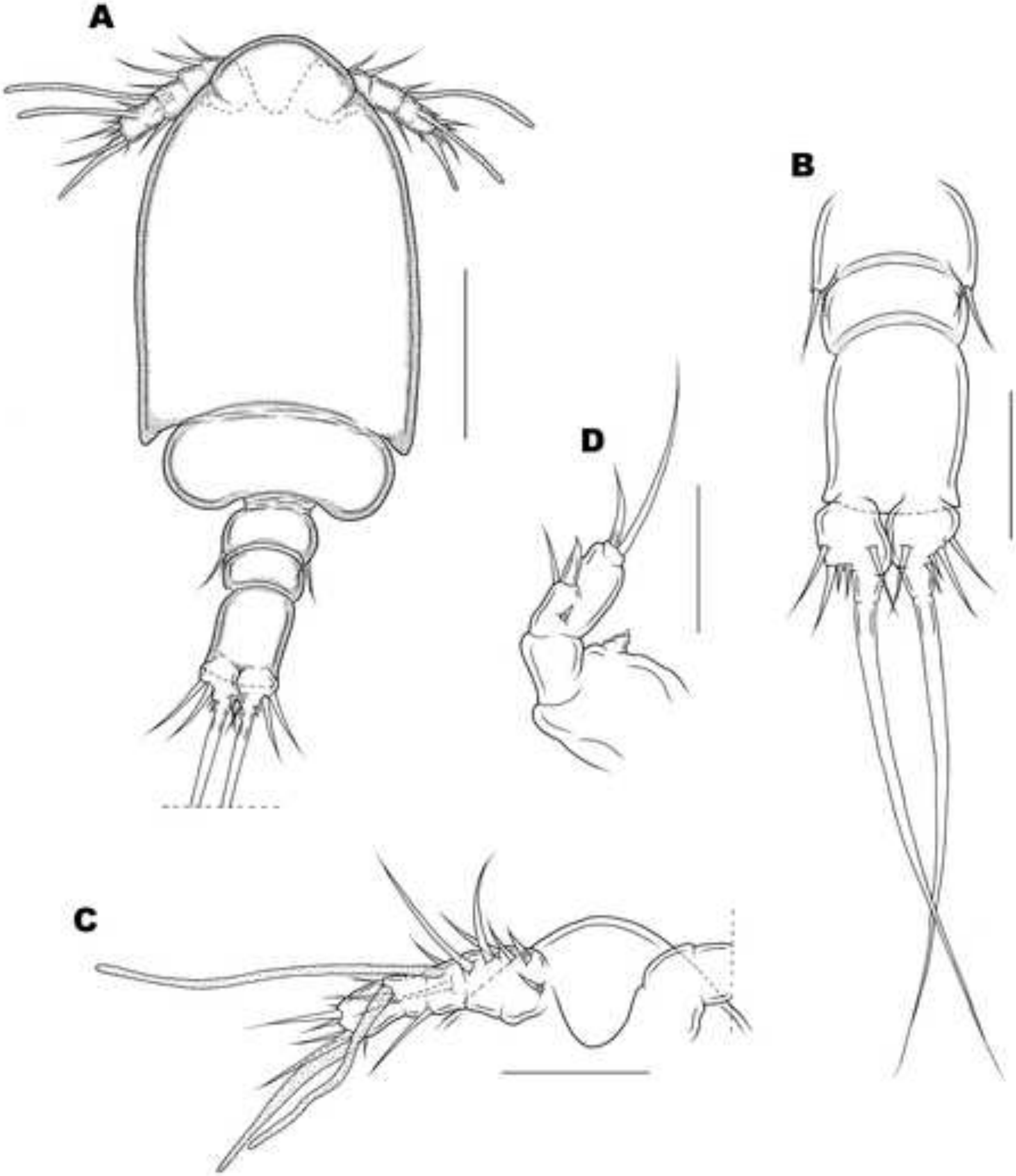

Figure 8

Click here to access/download;Figure;Figure8.tif

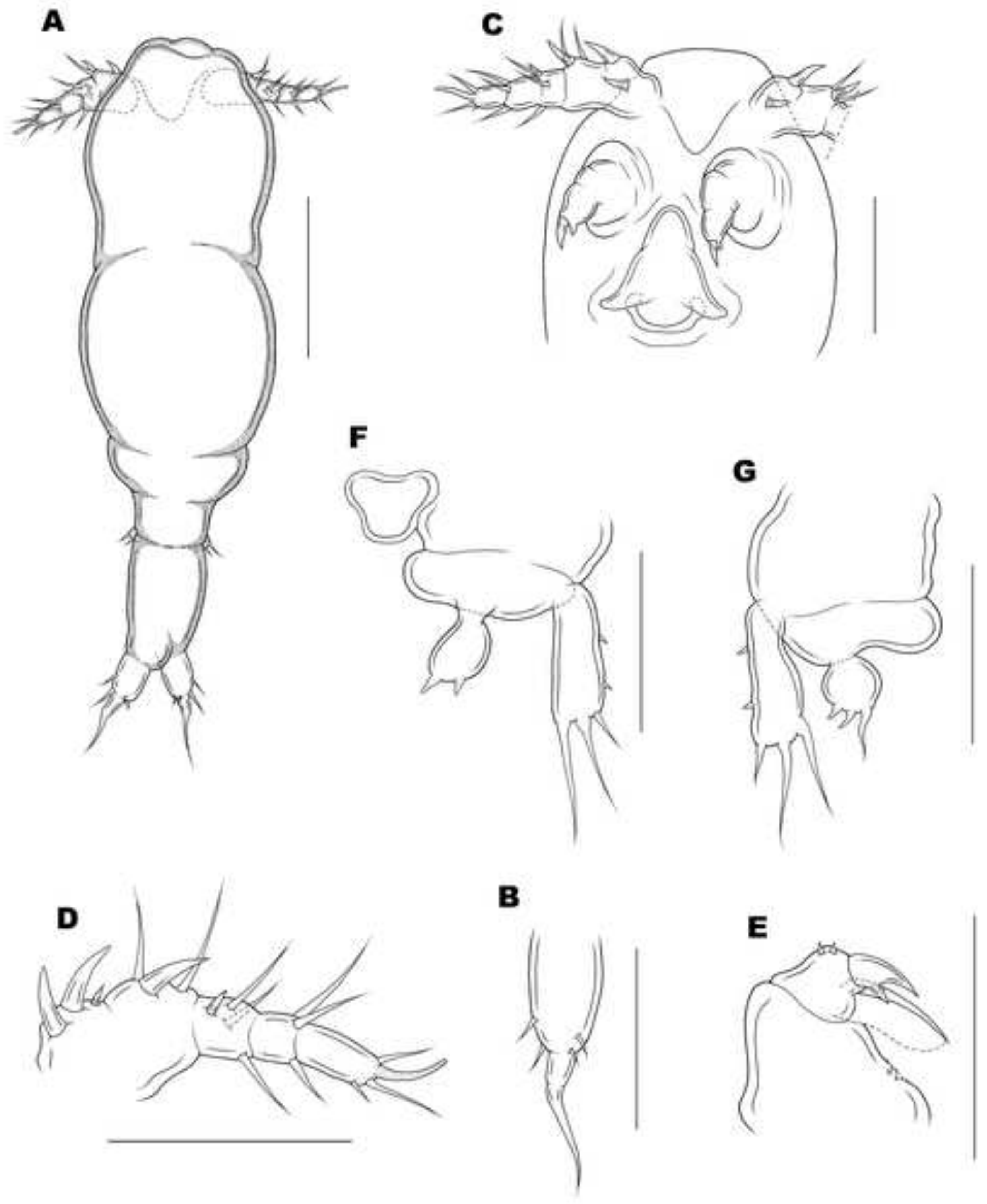

 Click here to access/download;Figure;Figure9.tif

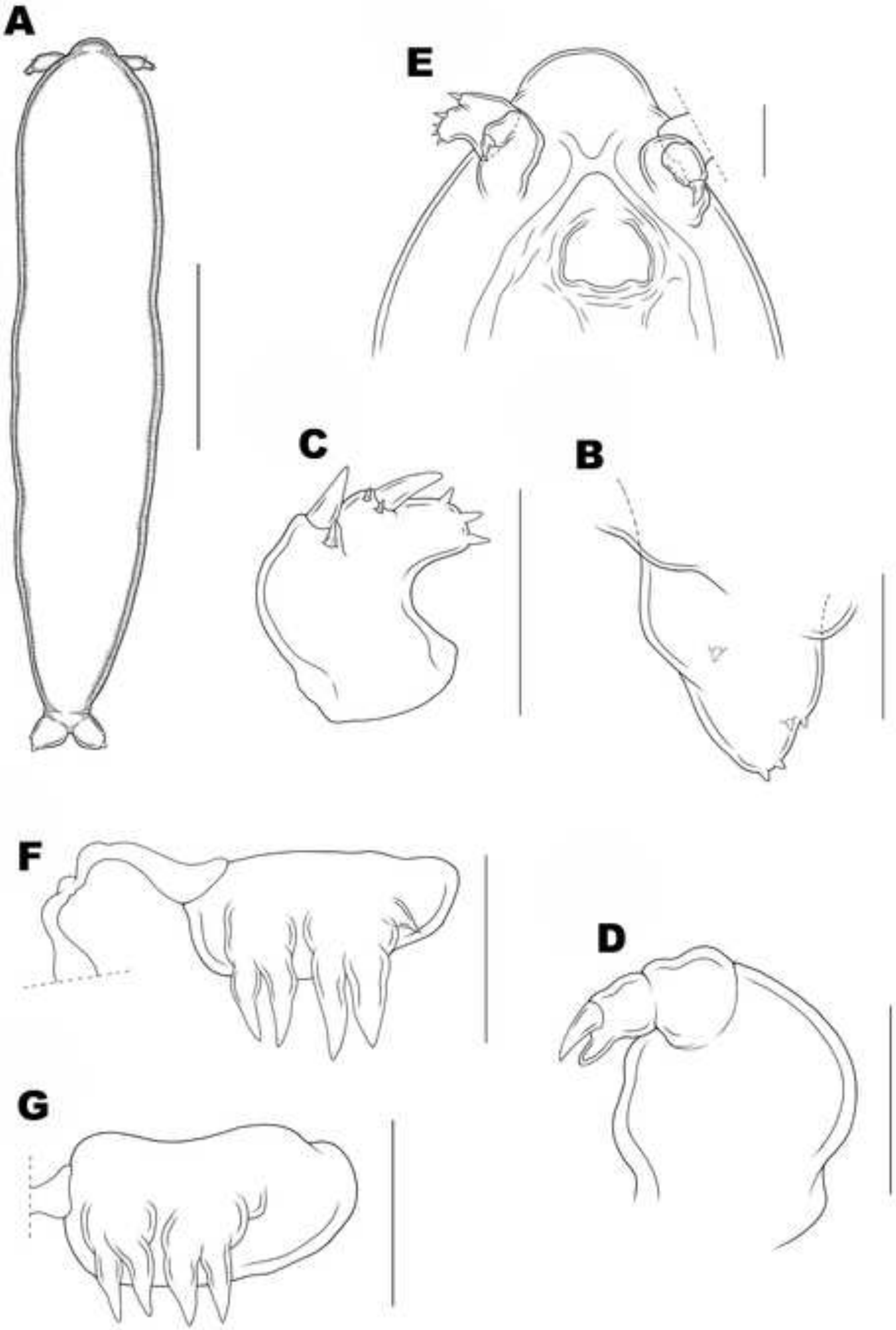

Figure 10

Click here to access/download;Figure;Figure10.tif

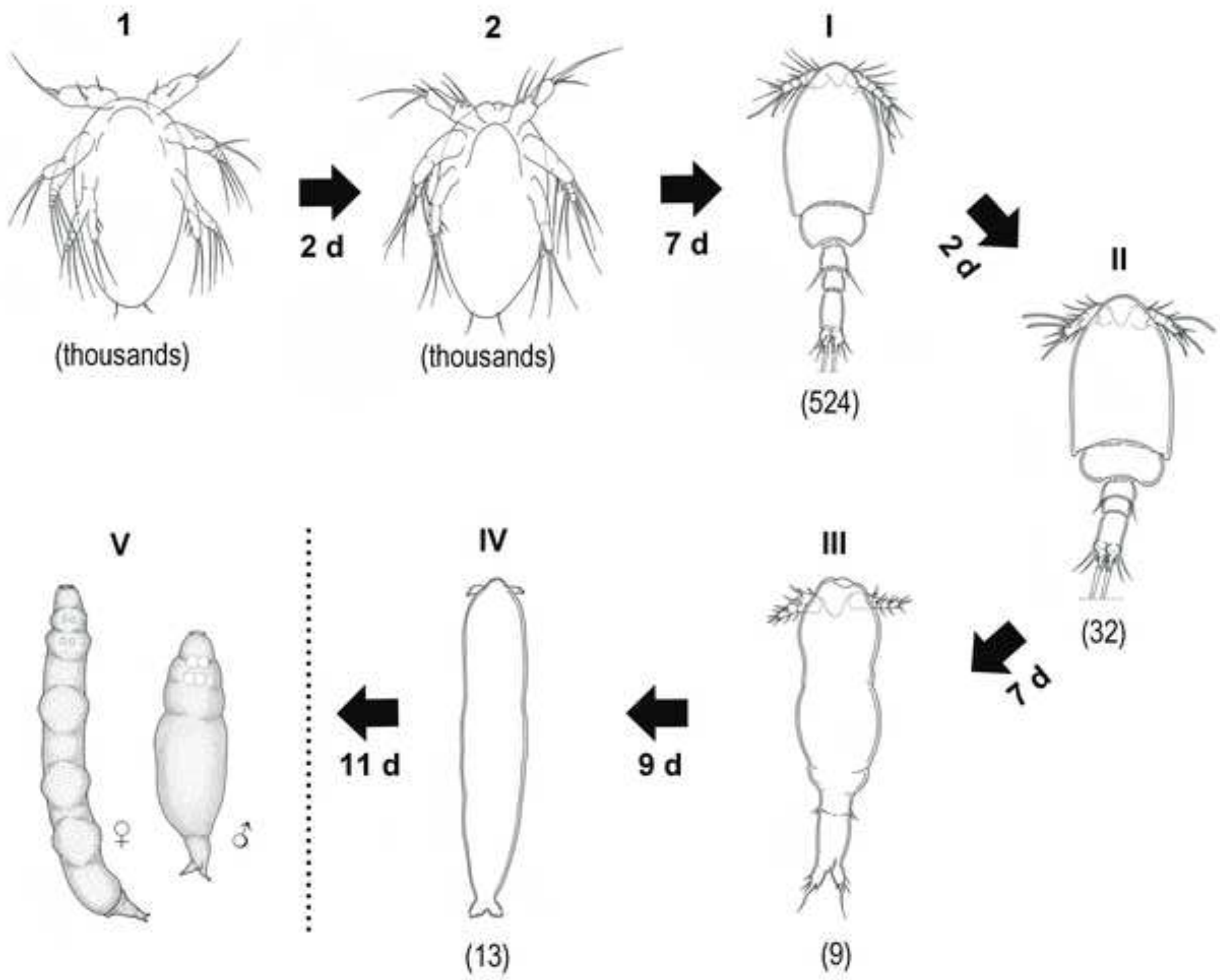

Supplement: Attachment — Submitted filename: PONE-D-22-29055_reviewer.pdf [file pone.0281013.s001.pdf]
